# Characterization of the RofA regulon in the pandemic M1_global_ and emergent M1_UK_ lineages of *Streptococcus pyogenes*

**DOI:** 10.1099/mgen.0.001159

**Published:** 2023-12-20

**Authors:** Xiangyun Zhi, Ana Vieira, Kristin K. Huse, Paulo J. Martel, Ludmila Lobkowicz, Ho Kwong Li, Nick Croucher, Ivan Andrew, Laurence Game, Shiranee Sriskandan

**Affiliations:** ^1^​ Department of Infectious Disease, Imperial College London, London, UK; ^2^​ Centre for Bacterial Resistance Biology, Imperial College London, London, UK; ^3^​ NIHR Health Protection Unit in Healthcare-associated Infection and Antimicrobial Resistance, Imperial College London, London, UK; ^4^​ CINTESIS@RISE, University of Algarve, Faro, Portugal; ^5^​ MRC Centre for Global Infectious Disease Analysis, Sir Michael Uren Hub, White City Campus, Imperial College London, London,, UK; ^6^​ Genomics Facility, UKRI-MRC London Institute for Medical Sciences (LMS), Imperial College London, London, UK

**Keywords:** transcriptomics, mga, standalone regulator, streptococcal pyrogenic exotoxin A, scarlet fever, Group A *Streptococcus*

## Abstract

The standalone regulator RofA is a positive regulator of the pilus locus in *Streptococcus pyogenes *. Found in only certain *emm* genotypes, RofA has been reported to regulate other virulence factors, although its role in the globally dominant *emm*1 *S. pyogenes* is unclear. Given the recent emergence of a new *emm*1 (M1_UK_) toxigenic lineage that is distinguished by three non-synonymous SNPs in *rofA*, we characterized the *rofA* regulon in six *emm*1 strains that are representative of the two contemporary major *emm*1 lineages (M1_global_ and M1_UK_) using RNAseq analysis, and then determined the specific role of the M1_UK_-specific *rofA* SNPs. Deletion of *rofA* in three M1_global_ strains led to altered expression of 14 genes, including six non-pilus locus genes. In M1_UK_ strains, deletion of *rofA* led to altered expression of 16 genes, including nine genes that were unique to M1_UK_. Only the pilus locus genes were common to the RofA regulons of both lineages, while transcriptomic changes varied between strains even within the same lineage. Although introduction of the three SNPs into *rofA* did not impact gene expression in an M1_global_ strain, reversal of three SNPs in an M1_UK_ strain led to an unexpected number of transcriptomic changes that in part recapitulated transcriptomic changes seen when deleting RofA in the same strain. Computational analysis predicted that interactions with a key histidine residue in the PRD domain of RofA would differ between M1_UK_ and M1_global_. RofA is a positive regulator of the pilus locus in all *emm*1 strains but effects on other genes are strain- and lineage-specific, with no clear, common DNA binding motif. The SNPs in *rofA* that characterize M1_UK_ may impact regulation of RofA; whether they alter phosphorylation of the RofA PRD domain requires further investigation.

## Data Summary

All analysed data are included in the supplementary Excel tables. Illumina RNA sequencing data have been submitted to the European Nucleotide Archive (ENA, www.ebi.ac.uk/ena) project PRJEB62819 with the accession numbers given in Table S1, available in the online version of this article.

**Impact Statement**

RofA belongs to the group of ‘mga-like’ bacterial regulatory proteins that comprise a DNA binding domain as well as a phosphorylation domain (PRD) that is responsive to changes in sugar availability. In certain *emm* genotypes of *Streptococcus pyogenes *, *rofA* sits upstream of the pilus locus, to act as a positive regulator. The recent emergence of an SpeA exotoxin-producing sublineage of *emm*1 *S. pyogenes* (M1_UK_) has focused attention on the role of RofA; M1_UK_ and its associated sublineages are characterized by three non-synonymous SNPs in *rofA* that include adjacent SNPs in the PRD. Here, we determine the impact of *rofA* deletion and the three *rofA* SNPs in both the widely disseminated M1_global_ clone and the newly emergent M1_UK_ clone. While production of SpeA undoubtedly contributes to infection pathogenesis, the evolution of M1_UK_ points to a role for metabolic regulatory rewiring in success of this lineage.

## Introduction

Resurgence of scarlet fever and invasive *Streptococcus pyogenes* infection in England has been associated with a sub-lineage (M1_UK_) of the pandemic M1T1 clone, which is distinguished from other prevalent *emm*1 strains by just 27 SNPs in the core genome, and increased expression of the phage-encoded superantigen SpeA [1]. Enhanced fitness of M1_UK_ is inferred from its expansion within the population of *S. pyogenes* in England, being detected first in 2010 to becoming dominant by 2015–2016.

Among the 27 M1_UK_ lineage-defining SNPs, three nonsynonymous mutations were identified in the gene *rofA* encoding the standalone transcriptional regulator RofA, a member of the RALP (RofA-like proteins) transcription regulator family [2]. The SNPs in RofA were also found in the two intermediate *emm*1 sublineages M1_13SNP_ and M1_23SNP_ that accompanied emergence of M1_UK_; M1_13SNP_ was detected as early as 2005, but did not make excess SpeA [3], consistent with a role for a SNP in the leader sequence of *ssrA* that contributes to the SpeA phenotype, and that is absent in the M1_13SNP_ strains [4].

RofA and its homologue Nra regulate genes of the fibronectin-binding, collagen binding, T-antigen (FCT) region [5]. RofA was first described as a positive transcriptional regulator of the fibronectin binding protein (*prtF*) in *emm6 S. pyogenes* [6]. Reported to bind specific motifs in target promoter DNA, RofA autoregulated its own transcription and appeared to positively regulate the *speB* operon as well as the pilus locus [7]. However, RofA has also been reported to negatively regulate a number of other regulators or virulence factors in an *emm* type-specific manner, such as *mga, sagA* and *speA*. RofA negatively regulated *speA* in *emm*6 *S. pyogenes* [2], but this was not the case for *emm*2 *S. pyogenes* [2, 8].

Despite intense genomic interrogation of the *emm1* lineage and increasing understanding of the genome-wide impact of two component regulators such as covRS (csrRS), little is known about the role of RofA in the *emm*1 lineage. Unlike other genotypes, *prtF* is absent in *emm*1 strains*,* which have a type 2 FCT region, wherein *rofA* is adjacent to the FCT region [9]. The purpose of this study was therefore to characterize the *rofA* regulon in *emm*1 *S. pyogenes* and determine the significance, if any, of the three SNPs in RofA detected in the new sub-lineage M1_UK_.

## Methods

### Bacterial strains and growth conditions

*S. pyogenes* strains were all non-invasive, clinical upper respiratory tract infection isolates that were collected in north-west London, UK, for the purpose of sequential genotyping and genome sequencing [1]. Isolates for RNA sequencing (RNAseq) were either in the M1_global_ or in the M1_UK_ lineage, excluding any intermediates; possessed a phage containing the *speA* gene; were wild-type for *covRS*; and had overall phage and superantigen content matching the reference *emm*1 sequence MGAS5005. Strains used in this study are listed in Table S1 and were previously used for transcriptome comparison of M1_global_ and M1_UK_ lineages [3].

Routinely, *S. pyogenes* were cultured on Columbia blood agar (CBA) (OXOID), Todd-Hewitt (TH) agar or in TH broth (OXOID) at 37 °C with 5 % CO_2_ for 16 h. Chemically defined medium (CDM) supplemented with different sugars (glucose or mannose) was also used for growth of *S. pyogenes *. Where appropriate, spectinomycin (50 µg ml^−1^) or kanamycin (400 µg ml^−1^) were added to the culture medium for *S. pyogenes. Escherichia coli* strains Top10 (Invitrogen) and DH5α were used for cloning and grown in Luria broth (LB) or on LB agar with kanamycin (50 µg ml^−1^) or spectinomycin (50 µg ml^−1^).

### Whole blood bacterial survival assay

Whole heparinized human blood was used in Lancefield assays as previously described [10]. Briefly approximately 50 c.f.u. of *S. pyogenes* was inoculated into 300 µl whole blood and incubated at 37 °C, with rolling for 3 h; each strain was tested in three normal donor whole blood samples (with technical triplicates for each donor). Blood donors whose samples permitted at least 2-fold multiplication of *S. pyogenes* over the 3 h incubation period were included. Input and output colony-forming units were measured by plating and the multiplication factor was calculated. Whole heparinized human blood was from consenting normal donors from a subcollection of the Imperial Tissue Bank (ICHTB, approved by Wales REC reference 17/WA/0161).

### Construction of *rofA* disruption mutant

A 491 bp fragment downstream of the *rofA* gene was amplified (forward primer: 5′-GGAATTCCTCTTACATAAGATTCATATC-3′, reverse primer: 5′-GGGGTACCCTCTTCCTACACTTAGAAAGC-3′) incorporating EcoRI and KpnI restriction sites into the 5′ and 3′ ends respectively, and cloned into the suicide vector pUCMUT. A 523 bp fragment upstream of the *rofA* gene was amplified (forward primer: ACGCGTCGACCGCCATGTCACCACATTGCG-3′, reverse primer: 5′-AACTGCAGGGGTTACCTGTGCCATAATC-3′) incorporating PstI and SalI restriction sites into the 5′ and 3′ ends respectively and cloned into the same plasmid. The final construct (pUCMUT_rofAKO_) was introduced into M1_global_ strains (H1488, H1489 and H1499) and MI_UK_ strains (H1491, H1496 and H1490) by electroporation and crossed into the chromosome by homologous recombination. Transformants were selected using kanamycin (400 µg ml^−1^). Successful disruption of the *rofA* gene and insertion of the kanamycin resistance cassette was confirmed by PCR, DNA sequencing and whole genome sequencing.

### Complementation of *rofA* strains

The *rofA* coding sequence, including native promoter, was amplified from both M1_global_ and M1_UK_ *S. pyogenes* DNA (forward primer: 5′-GACGCATGCCTCCTCTCAATGTGACATC-3′, reverse primer: 5′-ACGGATCCGTGGTGACATGGCGCTTATGTT-3′) incorporating BamHI and SphI restriction sites to each end of the PCR products, and cloned into BamHI- and SphI-digested shuttle vector pDL278 resulting in plasmids pDL_rofAM1_ and pDL_rofAM1UK_, which were confirmed by Sanger sequencing. The resulting plasmids were introduced into M1_globalrofAKO_ (H1561) and M1_UKrofAKO_ (H1582) by electroporation and selection using spectinomycin. The successful introduction of plasmid was confirmed by PCR specific for pDL278 backbone (forward primer: 5′-CATTCAGGCTGCGCAACTG-3′, reverse primer: 5′-TCGAATTCACTGGCCGTCG-3′) in each of the resulting isogenic strains H1591 (M1_globalrofAKO_ complemented) and H1592 (M1_UKrofAKO_ complemented).

### Construction of *rofA* three SNP mutant strains

To introduce the three SNPs in *rofA* typical of M1_UK_ into M1_global_ (H1488), a 597 bp fragment downstream of the *rofA* gene was amplified from M1_UK_ (H1496) template DNA (forward primer: 5′-AACTGCAGAGCACATTAAGTCCGATTGCAG-3′, reverse primer: 5′-ACGCGTCGACCCACACCTTAACTTAATCCCGA-3′) incorporating PstI and SalI restriction sites, and cloned into the suicide vector pUCMUT. A 1548 bp fragment upstream of the *rofA* gene was then amplified (forward primer: 5′-GGGGTACCCTAAAGTCGCGCAATGTGGTG-3′, reverse primer: 5′-AGCGAATTCGTGTAGGAAGAGAGGTCCCT-3′), incorporating EcoRI and KpnI restriction sites, and cloned into the same plasmid, resulting in pUCMUT_+rofA3snp_. The construct was introduced into M1_global_ (H1488) by electroporation and crossed into the chromosome by homologous recombination. Transformants were selected using kanamycin (400 µg ml^−1^). Successful allelic replacement and insertion of the kanamycin resistance cassette was confirmed by PCR, DNA sequencing and whole genome sequencing.

To fix the *rofA* three SNPs in M1_UK_ (H1496), the process was repeated to create plasmid pUCMUT_rofA3snpFIX_ however template DNA from M1_global_ (H1488) was used. The construct was introduced into M1_UK_ (H1496) by electroporation and crossed into the chromosome by homologous recombination. Transformants were selected using kanamycin (400 µg ml^−1^). Successful allelic replacement and insertion of the kanamycin resistance cassette was confirmed by PCR and DNA and whole genome sequencing.

### Quantitative real-time PCR

The extraction of RNA was done as described previously [11]. *S. pyogenes* were grown in THY broth until late logarithmic growth phase. The bacterial cultures were treated with aqua-phenol and phenol/chloroform/isoamyl alcohol (Sigma), and then precipitated with 2-propanol, followed by DNase treatment with TurboDNAfree (Ambion). RNA was reverse transcribed into cDNA using Transcriptor reverse transcriptase kit (Roche Diagnostics). Quantitative real-time PCR (qRT-PCR) was carried out for Spy0107, Spy0109 and *speA* using a real-time PCR System (Thermo Fisher Scientific) [3] and expression data were normalized to that of the housekeeping gene *proS* using a standard curve method as described previously [12].

### Transcriptome (RNAseq) analysis

*S. pyogenes* were grown in THY broth until late logarithmic growth phase. Three M1_global_ vs three M1_global RofAKO_, three M1_UK_ vs three M1_UKRofAKO_ were grown in triplicate (Table S1), RNA was prepared as previously described [11], and the quality and quantity of total RNA was evaluated using an Agilent 2100 Bioanalyzer with the RNA 6000 Nano Total RNA Kit. rRNA was depleted using a NEBNext rRNA Depletion Kit (Bacteria), and RNAseq next-generation sequencing (NGS) libraries were made using the NEBNext Ultra II Directional RNA Library Prep Kit for Illumina according to the manufacturer’s instructions, by the MRC Genomics Core Lab. A minimum of 10 million paired-end 100 bp reads were generated for each sample on HiSeq 2500 and NextSeq 2000 Illumina sequencers. Illumina sequencing data have been submitted to the European Nucleotide Archive (ENA, www.ebi.ac.uk/ena) project PRJEB62819 with the accession numbers given in Table S1. RNAseq data were analysed according to the followed pipeline. Read quality was accessed using FastQC (https://www.bioinformatics.babraham.ac.uk/projects/fastqc/), filtered and trimmed using trimmomatic [12], and mapped against the MGAS5005 (CP000017) reference genome using bowtie2 [13], with the highest sensitivity options. The resulting alignments were converted to sorted BAM files using vcftools [14]. Initial visualizations of the sequencing mapping were performed using the Integrative Genomics Viewer (IGV) [15], including the confirmation of *rofA* disruptions, three SNPs insertions and *emm*1 lineages. The mapped RNAseq reads were then transformed into a fragment count per gene per sample using HT-seq [16] packages and featureCounts [17] and the main results were compared. Exploratory data analysis (principal component analysis and heatmap of sample-to-sample distances) of the RNAseq data was implemented and plotted using the DESeq2 package [18]. Differential expression analysis in each dataset was performed using three different R packages (DESeq2 [18], EdgeR [19] and limma (https://bioconductor.riken.jp/packages/3.0/bioc/html/limma.html)) with a log_2_fold change of 0.5 and *P*<0.01 and *P*_adj_<0.05. Only differentially expressed (DE) genes in two of the three software programs used were considered as DE genes and used in the following analysis. Prophage regions were predicted using phaster [20], and curated by visual assessment and blast alignment. Operon prediction was performed using SP119 annotation [21] and motifs analysis was performed using XTREME (https://meme-suite.org/meme/doc/xstreme.html). Correlation coefficients for RNAseq were determined by plotting the log_2_ value of the array on the *x*-axis to the log_2_ value of the qRT-PCR on the *y*-axis. Linear regression was used to determine the line of best fit, and the resulting *R* ^2^ value was calculated, which represented the fitness of the data.

### Murine intranasal infection

FVB/n female mice 6–8 weeks old (Charles River) were briefly anaesthetized with isofluorane and challenged intranasally with 5×10^6^ c.f.*u. S. pyogenes *, administered as 5 µl per nostril. Nasal shedding was longitudinally and noninvasively monitored daily for 7 days following intranasal challenge using a nose-pressing technique [22]. Briefly, murine noses were pressed gently onto a CBA plate (Oxoid) 10 times, every 24 h. The resulting exhaled moisture was spread over the plate and colonies were counted following incubation at 37 °C, with 5 % CO_2_, for 24 h. On day 7 mice were killed, and nasal tissues, spleen and liver were dissected and plated onto CBA to quantify nasal and systemic *S. pyogenes* burden. Isogenic strains were compared over 7 days. To detect airborne shedding of bacteria within cages of infected mice, CBA settle plates (four per cage) were placed face up on the upper rack of individually HEPA filtered cages for 4 h on days, 0, 1, 2 and 3 post-infection as previously reported [22]. Airborne dispersal of *S. pyogenes* was quantified, by counting beta haemolytic colonies following overnight incubation of plates at 37 °C, with 5 % CO_2_. Procedures were in accordance with a UK Home Office Licence and local ethical review board approvals.

### Comparative model building and histidine location predictions

No homologues of Spy0106 *rofA* were found in Protein Data bank (PDB) sequences of known structure. Therefore, more sensitive methods for the detection of weak homologues were required. Two different approaches were used: an Hidden Markov Model (HMM)-based search using HH pred [23] and the threading server LOMETS (Zhang Lab) [24], both consistently leading to the identification of three PDB sequences (PDB codes 4R6I, 5WAY and 3SQN). The signature motifs identified in the three proteins were confirmed with an HMMER search [25]. Structural alignment and search of the three identified structures using the FATCAT flexible alignment server [26] confirmed their homology at the structural level, which suggests that any of them could be used as a template for model generation. 4R6I (AtxA protein, avirulence regulator from *Bacillus anthracis *) was the chosen template, as it had the longest alignment coverage of the target and the highest significance for the detected motifs. Using this template, models for M1_rofAKO_ and M1_UKrofAKO_ were produced using the MODELLER software [27]. The MODELLER template–target (guide) alignment was generated with the HHPRED server, and 50 models were produced for each of the *rofA* variants, using the recommended MODELLER parameters for a thorough structure search and minimization (MODELLER Manual, v 10.1). The quality of the generated models was assessed with the Ga341 score [28], the QMEAN score [29] and the MOLPROBITY server [30]. Each set of 50 models was loaded and visualized in the molecular viewer software PyMOL (Schrodinger) to access structural variance and the potential impact of amino acid substitutions between variants.

### Statistical analysis

All statistical analyses were performed with GraphPad Prism 5.0. Comparison of two datasets was carried out using an unpaired Student’s t-test and three or more data sets were analysed by Kruskal–Wallis tests followed by Dunn’s multiple comparison test or ANOVA and Bonferroni post-test depending on sample size. Survival data were analysed by a Mantel–Cox (log rank) test. A *P*-value of ≤0.05 was considered significant.

## Results

### Characterization of the RofA regulon in *emm*1 *S. pyogenes*

To systematically characterize the *rofA* regulon in *emm*1 *S. pyogenes,* where the FCT locus is structurally distinct from other genotypes [9, 31], and to determine the prevalence of strain-specific effects, *rofA* in-frame deletion mutants were constructed in three different *emm1* strains, H1488, H1489 and H1499 (each of which belong to the globally disseminated *emm*1 clade designated M1_global_), yielding the isogenic strains M1_H1488rofAKO_, M1_H1489rofAKO_ and M1_H1499rofAKO._

RNAseq analysis of broth-cultured isogenic strains of *S. pyogenes* identified 14 genes that were differentially expressed by at least 1.5-fold in all three isogenic strain pairs. Of these, 11 genes (78.57 %) were downregulated in all three of the M1_global_ RofA mutants, consistent with a predominant role for RofA as a positive regulator, while three genes (21.43 %) were upregulated (Table 1). As expected, transcription of genes associated with collagen binding and the pilus locus (Spy 0107–0114) was markedly downregulated. The effect of RofA mutation on Spy0107 and Spy0109 was confirmed in one panel of isogenic strains by qRT-PCR; mRNA transcripts were significantly reduced in the absence of RofA, and this was reversed by complementation (Fig. S1A and B). Spy1081 (PTS system, cellobiose-specific IIA component), Spy1281 (two-component response regulator) and Spy1453 (phage protein) were also downregulated in all three *rofA* mutants. Genes that were upregulated in all *rofA* mutants included Spy0212 (*N*-acetylmannosamine-6-phosphate 2-epimerase), Spy0213 (*N*-acetylneuraminate-binding protein) and *scpA*, the gene encoding C5a peptidase. RNAseq failed to show any impact of *rofA* deletion on *speA* transcription. To more rigorously establish that there was no effect of RofA on *speA*, we quantified the transcription of *speA* in M1_H1488rofAKO_ using qRT-PCR but observed no difference in comparison with the parent strain H1488 (Fig. S2A).

**Table 1.** Genes differentially regulated in all three M1_globalrofAKO_ strains compared with isogenic parent M1_global_ strains

Gene ID	Gene name	Description	H1488		H1489		H1499	
			log_2_fold change	*P*_adj_	log_2_fold change	*P*_adj_	log_2_fold change	*P*_adj_
Spy0106	*rofA*	Transcriptional regulator	−11.241	<0.001	−13.902	<0.001	−13.938	<0.001
Spy0107	na	–	−3.388	<0.001	−3.747	<0.001	−3.816	<0.001
Spy0108	na	Signal peptidase I	−3.348	<0.001	−3.510	<0.001	−3.952	<0.001
Spy0109	na	–	−3.288	<0.001	−3.514	<0.001	−3.983	<0.001
Spy0110	*eftLSL*	Putative exported protein	−3.478	<0.001	−3.399	<0.001	−4.363	<0.001
Spy0111	na	Hypothetical protein	−3.406	<0.001	−3.596	<0.001	−4.044	<0.001
Spy0113	na	Transposase	−2.674	<0.001	−2.463	0.001	−1.153	<0.001
Spy0114	na	Sortase	−2.248	<0.001	−1.710	<0.001	−0.612	0.002
Spy0212	na	*N*-Acetylmannosamine-6-phosphate 2-epimerase	0.723	0.002	1.855	0.001	1.417	<0.001
Spy0213	na	*N*-Acetylneuraminate-binding protein	0.812	0.002	1.936	0.026	1.506	<0.001
Spy1081	na	PTS system, cellobiose-specific IIA component	−0.755	0.019	−0.952	0.010	−1.270	<0.001
Spy1281	na	Two-component response regulator	−0.601	0.010	−0.579	0.031	−0.601	<0.001
Spy1453	na	Phage protein	−0.694	0.005	−0.676	0.033	−0.804	<0.001
Spy1715	*scpA*	C5A peptidase precursor	0.589	0.003	2.323	<0.001	0.510	0.010

na, Not applicable.

Mapping of transcripts used the MGAS5005 (CP000017) reference genome

The clear role of RofA in regulation of the pilus locus in the three different M1_global_ strains concealed quite marked interindividual differences between strains in the genes regulated by *rofA* (Tables S2–S4). Indeed, the number of genes downregulated by *rofA* mutation ranged from 60 to 146, while the number of genes upregulated ranged from 95 to 264 when considering individual isogenic strain pairs. In some cases, genes that were downregulated in one strain were upregulated in another. Notably only one *rofA* mutant showed upregulation of *speA* transcription, a derivative of H1499, which showed a 1.99-fold increase in *speA* transcription.

There were, however, some notable similarities in the *rofA* regulon between pairs of M1_global_ strains (Tables S5–S7). For example, when considering M1_global_ strains H1489 and H1499 (Table S6), streptolysin O and *nga* were upregulated in the absence of functional *rofA*, as were the two phage-encoded DNAses, Spd3 and SdaD2. However, the magnitude of effect was much greater in H1489; there was a 5–6-fold increase in transcription of phage-encoded DNAses in the absence of *rofA,* and an 8-fold increase in *slo* transcription.

When considering M1_global_ strains H1488 and H1499, we found that *rofA* mutation resulted in downregulation of the entire operon Spy1732-1736 comprising *speB* and genes concerned with its processing and export, as well as the adjacent gene encoding DNAseB. This amounted to a 9-fold reduction in *speB* in H1488 and a 3.2-fold reduction in *speB* in H1499. This effect of RofA on *speB* was not observed at all in the H1489 *rofA* mutant; indeed, in this strain *rofA* mutation led to a >2-fold increase in Spy1738 *spd* (DNaseB). This strain also demonstrated a convincing 3–4-fold upregulation of the entire SLS operon when *rofA* was mutated, alongside a 3–5-fold increase in genes that comprise the *mga* regulon including *scpA*, *sic* and *emm*. There was no obvious reason for the divergence between strains in the components of the *rofA* regulon; in particular, there were no obvious variants in known regulatory genes.

### Impact of three SNP RofA mutations in M1_global_ strains

There are three non-synonymous mutations in *rofA* that are characteristic of all of the recognized intermediate *S. pyogenes emm*1 sublineages that preceded or accompanied emergence of M1_UK_ [3]. To determine if these SNPs (M318I, F319V, D491N), two of which result in adjacent amino acid changes, have a measurable impact on *S. pyogenes* gene regulation, we introduced the same three SNPs into M1_global_ strain H1488 to generate GAS-M1_H1488rofA3SNPs_ (strain designation H1666). RNAseq was then used to compare the transcriptome of the transformant H1666 and the parent strain when cultured in broth in identical conditions to those above. Intriguingly, no genes were found to be differentially regulated using a threshold of log_2_ 1. Three genes were detected as DE with a log_2_ fold change of 0.5 (Spy0501; Spy1678; *smeZ*) but these did not overlap with transcripts affected by *rofA* deletion. Furthermore, there was no impact on pilus locus genes even when qRT-PCR was used (Fig. S1A and B).

Growth of the *rofA* mutant strains and those with the three SNPs introduced was evaluated in chemically defined medium with glucose or mannose as carbon sources, but differences identified between the *rofA* mutants and parent strains were minimal (Fig. S3A and B). We considered the possibility that deletion of *rofA* might impact growth of *S. pyogenes* in other more relevant media, but growth and survival of the three RofA mutant strains in whole human blood did not differ from the isogenic parent strains (Fig. S4).

As the main reservoir for *S. pyogenes* is the nasopharynx, we compared the ability of the panel of three isogenic *rofA emm*1 strains to cause experimental nasopharyngeal infection using an established mouse model. Detection of a difference in longevity of carriage of *emm*1 isogenic strains was challenging due to severity impacting group size over time despite the low inoculum volume. There was an apparent trend for mice infected with *S. pyogenes* carrying the *rofA* three SNPs (GAS-M1_H1488rofA3SNPs_) to cause more intense infection lasting up to 7 days (Fig. S5A–C).

### Characterization of the *rofA* regulon in emergent *S. pyogenes* lineage M1_UK_

We considered the possibility that the function of *rofA* may differ between *emm*1 lineages, and therefore undertook *rofA* gene deletion in a panel of three M1_UK_ strains (H1496, H1491 and H1490) to yield M1_H1496rofAKO_, M1_H1491rofAKO_ and M1_H1490rofAKO_. RNAseq comparison of the three pairs of isogenic strains cultured in broth revealed that 16 genes were differentially regulated in all three pairs of M1_UKrofAKO_ relative to the M1_UK_ parent strains (Table 2). Of these, 15 genes (93.8 %) were positively regulated by RofA, including the FCT locus genes, *bglA*2 operon (cellobiose PTS transporter operon), Spy1732 (protein export protein prsA precursor) and Spy1736 (hypothetical protein). A single gene, *glnQ*.2, was negatively regulated. Genes that were specifically DE in all three M1_UK_ strains (i.e. were not observed following *rofA* deletion in M1_global_ strains) included *epf*, *bglA2* operon, the *prsA* precursor and a hypothetical protein Spy1736.

**Table 2.** Genes differentially regulated in all three M1_UKrofAKO_ strains compared with parent M1_uk_ strains

Gene ID*	Gene name	Description	H1496		H1491		H1490	
			log_2_fold change*	*P*_adj_	log_2_fold change*	*P*_adj_	log_2_fold change*	*P*_adj_
Spy0106	*rofA*	Transcriptional regulator	−9.693	<0.001	−12.762	<0.001	−14.182	<0.001
Spy0107	na	–	−3.391	<0.001	−4.834	<0.001	−4.065	<0.001
Spy0108	na	Signal peptidase I	−3.220	<0.001	−5.301	<0.001	−4.389	<0.001
Spy0109	na	–	−3.486	<0.001	−5.181	<0.001	−4.517	<0.001
Spy0110	*eftLSL*	Putative exported protein	−3.305	0.001	−5.348	<0.001	−4.144	<0.001
Spy0111	na	Hypothetical protein	−3.622	<0.001	−5.206	<0.001	−3.966	<0.001
Spy0113	na	Transposase	−2.405	<0.001	−1.771	0.002	−2.179	0.002
**Spy0561**	***epf***	**Putative extracellular matrix binding protein**	**−0.851**	**0.002**	**−0.586**	**0.008**	**−1.038**	**0.010**
**Spy1077**	***glnQ.2***	**Glutamine transport ATP-binding protein**	**0.796**	**0.022**	**0.771**	**0.001**	**1.334**	**0.005**
**Spy1079**	**na**	**PTS system 2C cellobiose-specific IIC component**	**−1.572**	**<0.001**	**−0.789**	**0.001**	**−0.994**	**0.004**
**Spy1080**	**na**	**Hypothetical protein**	**−1.275**	**0.002**	**−0.712**	**0.010**	**−1.108**	**0.006**
**Spy1083**	**na**	**Transcription antiterminator 2C BglG family**	**−1.116**	**0.002**	**−0.589**	**0.002**	**−0.943**	**0.003**
**Spy1084**	**na**	**Outer surface protein**	**−1.099**	**<0.001**	**−0.821**	**0.003**	**−1.030**	**0.002**
**Spy1085**	***bglA.2***	**Beta-glucosidase**	**−1.218**	**0.014**	**−0.758**	**0.005**	**−0.996**	**0.004**
**Spy1732**	**na**	**Protein export protein prsA precursor**	**−1.518**	**0.025**	**−0.722**	**0.002**	**−0.789**	**0.003**
**Spy1736**	**na**	**Hypothetical protein**	**−2.023**	**0.004**	**−1.420**	**0.001**	**−0.688**	**0.028**

*Differentially expressed genes that are specific to M1_UK_ are in bold type.

na, Not applicable.

Considering the individual pairs of isogenic strains, *rofA* deletion led to downregulation of 73–124 genes in the three different M1_UK_ strains, and upregulation of 46–174 genes (Tables S8–S10). Although there was much interindividual strain variation among the M1_UK_ strains tested, there were again additional notable similarities in the *rofA* regulon between pairs of M1_UK_ strains (Tables S11–S13). Two M1_UK_ strains (H1496 and H1491, Table S11) showed 2–5-fold upregulation of streptolysin O expression and downregulation of *speB* and adjacent genes following *rofA* disruption, similar to results observed among M1_global_ strains. RofA inactivation in the two M1_UK_ strains H1496 and H1490 (Table S12) resulted in downregulation of two operons: the V-type sodium ATP synthase *ntp* operon, and the citrate lyase *cit* operon. Neither operon was downregulated among M1_global_ strains. Interestingly, RofA inactivation in M1_UK_ strain H1491 had an opposing effect on both the *ntp* operon and citrate lyase operon, both of which were upregulated in response to *rofA* mutation, along with upregulation of the *pur* operon (Spy0022-0034), again illustrating quite marked interindividual strain variation in the impact of *rofA* mutation even among strains that are seemingly phylogenetically related.

There was a substantial overlap in seven genes that were positively regulated by RofA in both M1_global_ and M1_UK_ lineages, all of which were part of the FCT locus operon. Gene expression differences were confirmed by qRT-PCR (Fig. S1). We conclude that these genes therefore represent the core RofA regulon in *emm*1 *S. pyogenes* (Table 3). Similar to earlier findings, there was no evidence that *speA* was regulated by RofA in the M1_UK_ lineage, as confirmed by qRT-PCR (Fig. S2B). Taken together the results pointed to a diversified RofA regulon in M1_UK_ strains. Analysis of promoter regions of genes regulated by RofA in at least two of the three strains of each lineage (M1_UK_ and M1_global_) was performed and no common motif was identified, even when genes up- and downregulated were considered separately (Fig. S6).

**Table 3.** RofA core regulon: genes differentially expressed in both M1_globalrofAKO_ and M1_UKrofAKO_ strains compared with parent M1_global_ and M1_UK_ strains

Gene ID	Gene name	Description	M1_global_						M1_UK_					
			H1488		H1489		H1499		H1496		H1491		H1490	
			log_2_fold change	*P*_adj_	log_2_fold change	*P*_adj_	log_2_fold change	*P*_adj_	log_2_fold change	*P*_adj_	log_2_fold change	*P*_adj_	log_2_fold change	*P*_adj_
Spy0106	*rofA*	Transcriptional regulator	-11.241	<0.001	−13.902	<0.001	−13.938	<0.001	−9.693	<0.001	−12.762	<0.001	−14.182	<0.001
Spy0107	na	–	−3.388	<0.001	−3.747	<0.001	−3.816	<0.001	−3.391	<0.001	−4.834	<0.001	−4.065	<0.001
Spy0108	na	Signal peptidase I	−3.348	<0.001	−3.510	<0.001	−3.952	<0.001	−3.220	<0.001	−5.301	<0.001	−4.389	<0.001
Spy0109	na	–	−3.288	<0.001	3.514	<0.001	−3.983	<0.001	−3.486	<0.001	−5.181	<0.001	−4.517	<0.001
Spy0110	*eftLSL*	Putative exported protein	−3.478	<0.001	−3.399	<0.001	−4.363	<0.001	−3.305	0.001	−5.348	<0.001	−4.144	<0.001
Spy0111	na	Hypothetical protein	−3.406	<0.001	−3.596	<0.001	−4.044	<0.001	−3.622	<0.001	−5.206	<0.001	−3.966	<0.001
Spy0113	na	Transposase	−2.674	<0.001	−2.463	0.001	−1.153	<0.001	−2.405	<0.001	−1.771	0.002	−2.179	0.002

Column headers provide parent (wild-type) strain; comparisons are with the cognate isogenic RofA deletion mutant. na, Not applicable.

### Reversal of the RofA three SNPs in M1_UK_

**Fig. 1.**
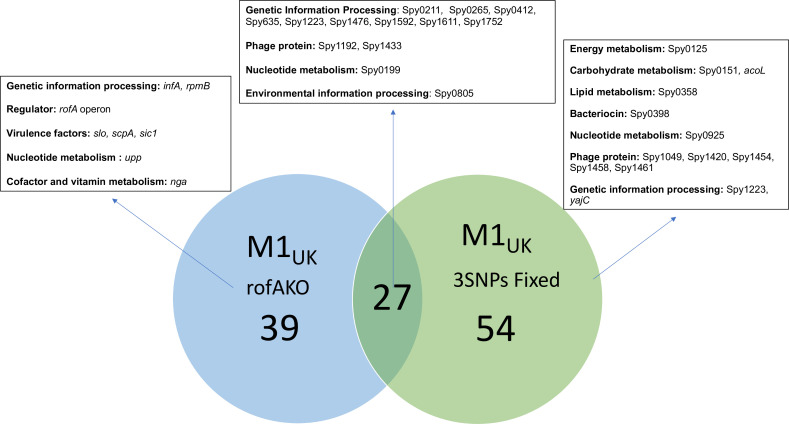
Venn diagram enumerating upregulated genes following disruption of *rofA* (blue circle, M1_UKrofAKO_ left) and fixing of three SNPs (green circle, M1_UK3SNPsfixed_, right) in isogenic derivatives of M1_UK_ strain H1496. Number of overlapping upregulated genes shown in centre. Representative genes assigned to each category are indicated in boxes.

As the RofA regulon in M1_UK_ appeared distinct from M1_global_, it seemed possible that the three SNPs present in *rofA* in the M1_UK_ lineage may be significant in this strain background, noting that the strain background is characterized by a number of additional SNPs that may alter strain physiology. We therefore evaluated the impact of ‘reversing’ the *rofA* three SNPs in M1_UK_ strain H1496, to result in strain M1_UKrofA3SNPsFixed_ (strain designation H1665). In contrast to our findings in M1_global_, where introduction of the three SNPs made little impact on the transcriptome, we found that 91 genes were differentially regulated in M1_UKrofA3SNPsFixed_. These genes included bacteriocin, *epuA*, eight phage genes and several hypothetical genes (Table S14). There was surprising overlap between the transcriptome of the RofA deletion mutant and the mutant with reversal of the three SNPs in the same strain background (Fig. 1), suggesting that the function of RofA, at least in this strain, was impaired by reversal of the three SNPs. Two genes (Spy1142-1143) were downregulated in both M1_UKrofAKO_ and M1_UKrofA3SNPs_. Over 40 % (27/66) (Table 4) of the genes upregulated in the same M1_UK_ *rofA* mutant were also upregulated when the three SNPs in *rofA* were ‘fixed’; these included three cytosolic proteins, two phage proteins, three large subunit (LSU) ribosomal proteins, *epuA*, as well as iojap protein family protein and *nrdG*. None of these genes were part of the core RofA regulon. The findings attribute a role for the three SNPs in RofA repressor activity in M1_UK_ strains.

**Table 4.** Genes differentially expressed in both M1_UKrofA3SNPsFixed_ and M1_UKrofAKO_ compared with parent M1_UK_ strain

Gene ID	Gene name	Description	M1_UKrofA3SNPsFixed_		M1_UKrofAKO_	
			log_2_fold change	*P*_adj_	log_2_fold change	*P*_adj_
Spy0073	na	Hypothetical protein	1.334	0.003	1.305	0.007
Spy0199	na	Deoxyuridine 5′-triphosphate nucleotidohydrolase	0.752	0.003	0.828	0.045
Spy0211	*rpmH*	LSU ribosomal protein L34P	1.483	0.015	1.669	0.003
Spy0265	na	iojap protein family	1.423	0.003	1.510	<0.001
Spy0269	na	Putative cytosolic protein	1.213	0.003	1.260	0.001
Spy0399	na	Putative membrane-associated protein	3.000	0.005	2.556	0.003
Spy0412	na	LSU ribosomal protein L33P	1.793	0.002	1.671	0.008
Spy0493	na	Putative cytosolic protein	1.494	0.002	1.154	0.011
Spy0585	*epuA*	epuA protein	1.392	0.003	1.176	0.002
Spy0635	*rpmA*	LSU ribosomal protein L27P	0.710	0.020	0.659	0.037
Spy0805	*srtK*	Nisin biosynthesis sensor protein	0.655	0.011	0.680	0.017
Spy0985	na	phnA protein	0.947	0.006	0.894	0.012
Spy1087	na	Putative cytosolic protein	1.889	0.005	2.208	<0.001
Spy1089	na	Hypothetical protein	2.783	0.001	2.150	0.005
Spy1192	*rsuA*	Phage protein	2.609	0.001	1.689	0.015
Spy1223	na	DNA-binding protein HU	1.011	0.004	0.991	0.015
Spy1239	na	Putative cytosolic protein	1.062	0.001	1.447	0.014
Spy1295	na	Putative cytosolic protein	0.814	0.016	1.084	0.015
Spy1433	na	Phage protein	1.361	0.004	1.286	0.007
Spy1476	na	ATP/GTP hydrolase	0.882	0.011	1.165	0.001
Spy1504	na	Putative membrane spanning protein	1.428	0.002	1.175	0.010
Spy1592	na	Ribosomal-protein-alanine acetyltransferase	0.612	0.032	0.691	0.015
Spy1611	*rpoE*	DNA-directed RNA polymerase delta chain	0.646	0.023	0.947	0.001
Spy1650	na	degV family protein	1.274	0.003	0.886	0.046
Spy1752	na	LSU ribosomal protein L33P	1.849	0.001	1.287	0.032
Spy1789	*nrdG*	Anaerobic ribonucleoside-triphosphate reductase activating protein	0.643	0.027	0.738	0.009
Spy1792	na	Hypothetical protein	1.293	0.003	1.368	0.008

na, Not applicable.

We considered the possibility that the activity of RofA may impact M1_UK_ *S. pyogenes* growth but did not detect a significant difference when comparing growth of a panel of isogenic strains in CDM with glucose or mannose as carbon sources (Fig. S3C and D). We again compared the survival of three M1_UK_ parent strains, with the three isogenic M1_UK_ *rofA* mutant derivatives in whole human blood, but found no difference between the isogenic strains (Fig. S4). Finally, we were unable to detect a difference when comparing the strains in a murine model of nasopharyngeal carriage albeit that, again, group sizes were affected by infection severity (Fig. S7A–C).

### Bioinformatic analysis of RofA and implications of the three SNPs

**Fig. 2.**
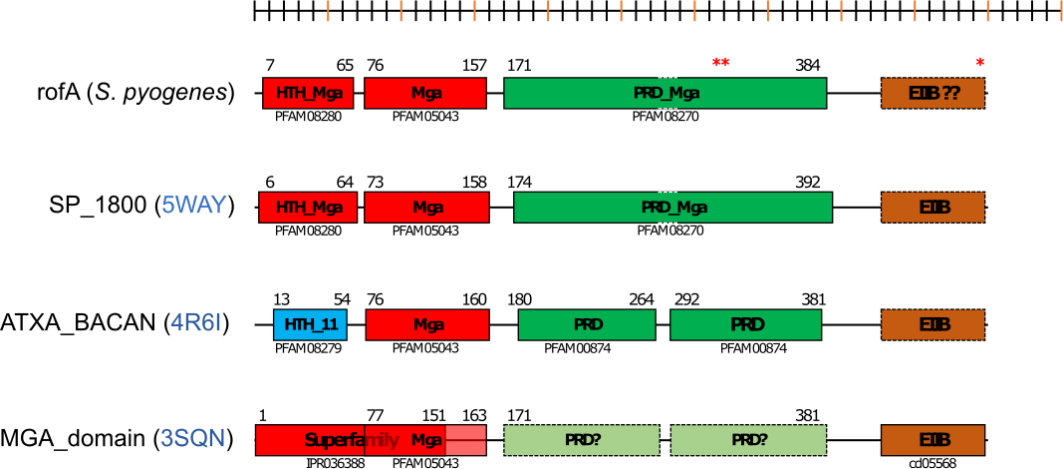
Schematic representation of RofA and *mga* homologous domain structures. Defined lines indicate domains that have been identified by their interpro signature. Blurred boundaries indicate domains predicted by structural similarity. Helix–turn–helix (HTH) and DNA-binding domains are represented in blue and red, phosphotransferase system regulatory domains (PRD) in green and putative EIIB domains in orange. From the top, the diagram depicts RofA; MgaSpn, an Mga regulator from *Streptococcus pneumoniae* (5WAY); AtxA, a virulence regulator from *Bacillus anthracis* (4R6I); and a putative Mga family transcriptional regulator from *Enterococcus faecalis* (3SQN). RofA amino acid changes in M1_UK_ are indicated by red asterisks.

**Fig. 3.**
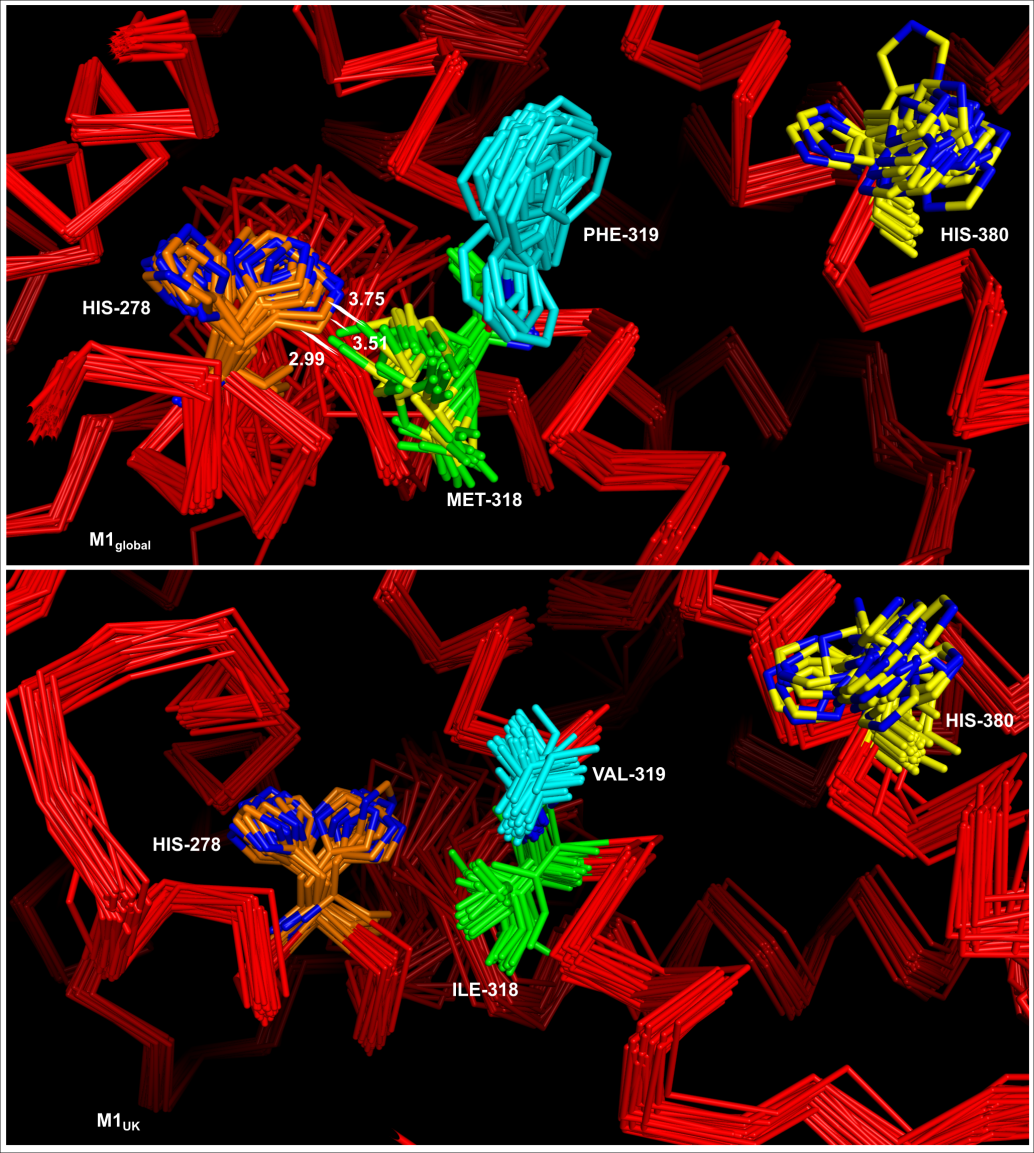
Conformations of His-278 and Met/Ile-318 in the 50 homology models of RofA in M1_global_ lineage strains (upper panel) and M1_UK_ lineage strains (lower panel). His-278 is represented in orange and Met/Ile318 residues in green. Interatomic distances between His278 and Met318 are represented by a line when they are below the van der Waals contact distance for the corresponding atoms. Allelic variants Phe/Val-319 shown in turquoise. More distant histidine residue His-380 is shown in yellow.

To predict if the location of the three nonsynonymous SNPs would have consequences for RofA regulatory function, bioinformatic analysis of the RofA amino acid sequence was carried out. Despite the low genetic similarity (and 19–20 % amino acid sequence identity), domain organization of RofA was remarkably similar to AtxA, a virulence regulator from *Bacillus anthracis* (4R6I); MgaSpn, an Mga regulator from *Streptococcus pneumoniae* (5WAY); and putative Mga family transcriptional regulator from *Enterococcus faecalis* (3SQN) as shown in Fig. 2. RofA contains two putative DNA binding domains: the helix–turn–helix mga domain (HTH_mga) (residues 7–65) and an mga DNA-binding domain (Mga) (76–156 residues), followed by a phosphotransferase system (PTS) regulation domain (PRD) of Mga located between residues 171 and 384 and a putative C-terminal PTS EIIB-like domain only identified by structural analysis. The two adjacent RofA amino acid changes in M1_UK_ and related sublineages are in the PRD_mga domain (M318I and F319V) while the other amino acid change (D491N) is in the final section of a putative EIIB-like domain. *In silico* analysis suggests that, unlike other mga-like proteins, RofA may have just one longer fused functional PRD instead of two. PRD_*mga* domains are crucial for *mga* activation, through histidine phosphorylation events, in response to environmental sugar status, while EIIB-like domains may influence dimerization [32–35]. To determine if the three RofA SNPs in M1_UK_ may have an impact on RofA histidine phosphorylation, 50 RofA model structures of each form were generated with the comparative modelling software MODELLER. Analysis highlighted a possible effect of the M318I substitution upon His278: the 50 superimposed M1_global_ models revealed that, in at least three different conformations, Met318 and His278 residues are close enough to produce steric hindrance (Fig. 3), with a probable mode of interaction (His N to Met S) that is well described [36]. By contrast, no such interaction can occur in M1_UK_ as Met318 is replaced by an isoleucine residue, with a side chain comprising only nonpolar carbon and hydrogen atoms (Fig. 3). Consequently, the distribution of His278–M318I distances are larger in the M1_UK_ than in M1_global_ models (Fig. S8), an effect that is further reinforced by the shorter length of the Ile chain in M1_UK_.

## Discussion

In this study, we analysed the RofA regulon in six different *emm*1 strains that were representative of the two contemporary major *emm*1 lineages (M1_global_ and M1_UK_). The transcriptome of RofA deletion mutants has not to our knowledge been fully reported; our results suggest that the transcriptomic changes may vary according to strain background, even within the same *emm* genotype. Although the pilus locus was a major target of RofA regulation in both lineages, RofA mutation led to a number of discrete transcriptomic changes that were unique to M1_UK_ strains and were not seen in M1_global_. Although introduction of the three SNPs into *rofA* made little or no impact in an M1_global_ strain, the reversal of three SNPs in an M1_UK_ strain led to an unexpected number of transcriptomic changes that, in part, recapitulated transcriptomic changes seen when deleting RofA. Computational analysis identified a key role for target residues in RofA that may interact with histidine residues, predicting that phosphorylation of RofA and function of the PRD may differ in M1_UK_ strains.

RofA was first identified as a positive regulator of the *prtF* genes in *emm*6 *S. pyogenes* [6]. Previous investigators have identified apparent serotype-specific differences in RofA target genes when evaluating *emm*2, *emm*6 and *emm*49 isogenic mutants; our data suggest that the range of target genes that are subject to RofA regulation may well vary from strain to strain despite sequence identity between promoters [2, 6, 37]. Based on our findings, the regulon of RofA cannot easily be explained by consensus DNA binding sequence motifs or involvement of other regulators. Indeed, the predicted motifs were quite different from previously published RofA motifs [38]. The sequence logos contained poly-AT tracts, which are known to be relatively flexible [39]. This suggests RofA may recognize DNA conformation or curvature, rather than a specific sequence, as proposed for the structurally similar AtxA protein [40]. This would explain how this single protein can have such a variable regulon across the species, and even between closely related isolates of the same *emm* type. Hence subtle changes to the protein’s structure, such as those caused by the three SNPs investigated in this study, may have an effect through differentially influencing RofA’s association with multiple loci, rather than simply changing its affinity for a clearly defined binding motif.

We have undertaken systematic analysis of the RofA regulon in the widely successful *emm*1 pandemic lineage (M1_global_). Although our findings confirm the role of RofA as a strong positive regulator of the pilus locus in *emm*1 strains as previously reported, we also identified a clear role for RofA in regulation of Spy0212/0213, Spy1081 (PTS system), Spy1281 (two-component regulator), phage protein (Spy1453) and *scpA*. RofA was reported to suppress transcription of *speA*, *sagA* and *mga* [2] but we could not confirm this in *emm*1 strains. In the current study, RNAseq identified *speA* as a target for repression in only one *rofA* mutant. Although single M1_global_ and M1_UK_ strains showed upregulation of *sagA* upon *rofA* deletion, other strains from each lineage showed downregulation of *sagA*, underlining the importance of considering more than one strain when examining regulatory gene control. When we compared the RofA regulon between M1_UK_ and M1_global_ we identified additional unique genes that were regulated in only M1_UK_ including *epf*, *glnQ.2*, the *bglA.2* operon, Spy1732 and Spy1736.

RNAseq data showed that two of the three M1_UK_ *rofA* mutant strains exhibited downregulation of the *ntpAB* operon that encodes V-type ATPases. Interestingly our recently reported proteomic data showed that strains from M1_UK_ and the M1_23SNP_ sublineage have reduced (rather than increased) levels of NtpA and B in the cytosol compared with strains from M1_global_ and the M1_13snp_ sublineage [3]. The role of the V-type ATPase in the physiology and pathogenesis of *S. pyogenes* is not known though the operon may be regulated by small RNAs [41]. V-type ATPases, located in the plasma membrane, couple the transfer of protons or sodium cations across the membrane with ATP hydrolysis or synthesis, and are responsible for cytoplasmic proton extrusion, regulating internal pH [42]. *S. pyogenes* performs only lactic acid fermentation for production of energy and can survive despite lowering the pH to ∼5.4 in growth medium [41]. Thus, the V-type ATPase in *S. pyogenes* might be involved in pumping hydrogen ions from the bacterial cytosol to overcome acid stress to survive the acidic conditions inside the host lysosome.

RofA also appears to play a role in positive regulation of the citrate lyase operon which is also involved in pH tolerance, in some, but not all, *emm*1 strains. One M1_global_ and two M1_UK_ *rofA* mutant strains showed reduced transcription in the *citABCDE* operon. Citrate lyase is a key enzyme that allows the microorganism to enter the citric acid cycle in the reductive mode and the citrate lyase operon may help *S. pyogenes* adapt to metabolic stress, such as low pH, lactate accumulation and nutrient-deprived, hypoxic conditions [43–45]. This metabolic ‘switch’ facilitates survival during environmental transitions encountered in the infective process [45], while absence of the citrate lyase operon may render *emm*12 *S *. *pyogenes* strains less fit under nutrient-deprived conditions [43].

The reversal of the three SNPs in *rofA* in an M1_UK_ strain resulted in a transcriptomic effect that in part emulated *rofA* deletion in the same strain. This suggests that the function of *rofA* in M1_UK_ strains is somehow reliant on these three SNPs, while this is not the case in M1_global_ strains, potentially because of the subsequent adaptive mutations that have occurred in M1_UK_. We noted upregulation of phage genes in the strain with reversal of the *rofA* three SNPs that was unexpected, and may reflect a stress response. Davies *et al*. recently reported the effects of reversal of the three 3SNPs in an M1_UK_ strain, and found that no gene was differentially expressed by the repair of the *rofA* three SNPs [4]. In contrast, we found 91 genes to be differentially regulated in the *rofA* repaired strain (10 genes downregulated and 81 genes upregulated). While this could be a strain-specific effect, or related to the different growth media used, or related to different expression thresholds used in each study, the data cannot be compared directly as the two studies used different reference strains.

Currently, the mechanism by which RofA is controlled is unknown. Experimental data relating to Mga (the closest related protein studied) suggests that the PRD is activated by histidine phosphorylation events that lead to a defect in protein oligomerization, altered gene expression and, in some cases, virulence attenuation [32–35]. The number and positions of phosphorylated histidines within the PRD_*mga* domain vary among the regulators, and phosphorylation can positively or negatively affect protein activity [32]. Our protein model predictions show that the amino acid substitutions between M1_global_ and M1_UK_ may interfere with RofA histidine phosphorylation. In fact, M318I replacement could disrupt a stabilizing interaction between this residue and His278. This native interaction could bias the His conformation towards the proximal rotamer (when the His residue is nearest to M318), limiting access to its kinase enzyme and resulting in different phosphorylation activation events between lineages. Whether the PRD of RofA is indeed phosphorylated at His278 is currently unknown, as is the experimental impact of the three SNPs on phosphorylation of RofA.

Our study has highlighted the importance of examining multiple strains when considering the role of *S. pyogenes* regulators, but a limitation is the number of strains that can be practically examined in different growth conditions. This extends in particular to the testing of strains *in vivo*, where comparison of multiple isogenic strains, or different lineages, in the same model can be challenging and may require a prohibitive number of mice per group to demonstrate a clear difference. Indeed, the pleiotropic transcripts affected by RofA deletion may affect *S. pyogenes* survival phenotypes in opposing directions, such that the net effect of RofA deletion may not recapitulate the effects of deletion of pilus protein genes [5]. The acquisition of the three SNPs in *rofA* in the M1T1 *S. pyogenes* lineage is unique to the *emm*1 intermediate sublineages and M1_UK_; to date, we have not detected these three SNPs in any other *emm* genotype. As such, the SNPs act as a useful marker of the new lineages. The three SNPs in *rofA* were identified as early as 2005 [3] and have persisted throughout the evolution of M1_UK_, present in both M1_13SNP_ and M1_23SNP_ strains, underlining a key role in the success of the novel lineage.

## Supplementary Data

Supplementary material 1

Supplementary material 2
